# Association of depression and resilience with fertility quality of life among patients presenting to the infertility centre for treatment in Karachi, Pakistan

**DOI:** 10.1186/s12889-020-09706-1

**Published:** 2020-10-23

**Authors:** Shireen Shehzad Bhamani, Nida Zahid, Wajeeha Zahid, Salima Farooq, Saima Sachwani, Marilyn Chapman, Nargis Asad

**Affiliations:** 1 School of Nursing and Midwifery, Aga Khan University, Karachi, Pakistan; 2 Department of Surgery, Aga Khan University, Stadium Road, Karachi, 74800 Pakistan; 3 Community Health Sciences Department Aga Khan University, Karachi, Pakistan; 4 Vancouver Island University, Vancouver, Canada; 5 Department of Psychiatry, Aga Khan University, Karachi, Pakistan

**Keywords:** Quality of life, Infertility, Gender, Resilience, Depression

## Abstract

**Background:**

In Pakistan there is a dire need to explore the quality of life in infertile males and females and its undesirable psychological outcomes. This, study aimed to compare the quality of life (QoL) of males and females visiting an infertility centre for treatment and to assess its association with resilience, depression, and other socio-demographic factors.

**Methods:**

An Analytical Cross-Sectional study was conducted amongst infertile males and females at the Australian Concept Infertility Medical Centre (ACIMC), Karachi, Pakistan. The non-probability (purposive) sampling strategy was used to recruit the participants. The sample size was 668. Data was analysed using STATA version 12. FertiQoL tool, Beck II Depression Inventory Tool and Resilience Scale 14 (RS-14) were used for assessing the quality of life, depression and resilience respectively of infertile patients.

**Results:**

Total 668 infertile patients, 334 males and 334 females participated in the study. The mean age was 35.53 ± 6.72, among males, and 30.87 ± 6.12 among females. The mean resilience scores were significantly higher among males, (77.64 ± 8.56), as compared to females (76.19 ± 8.69) (95% CI; − 2.757, − 0.1347). However, a significantly higher proportion of females were depressed (13.8%) as compared to males (6%). The mean QoL scores for the general health domain, emotional domain, mind and body domain, and relational domain, and the total QoL were significantly higher in males as compared to females (*p* value< 0.001); however, QoL for the social domain was not significantly different in both the groups. On multivariable linear regression resilience and depression among males had a significant association with QoL, after adjusting for the covariates educational status, monthly income, and number of friends. Similar association was observed among females after adjusting for the covariate monthly income only.

**Conclusion:**

Fertility related QoL of men and women has a significant association with no formal education, number of friends, income, depression and resilience. Therefore, health care professionals in the field of infertility must be adequately trained to respond to the needs of individuals going through these psychological problems.

## Background

Worldwide, infertility is recognized as a public health issue affecting the reproductive health of both genders [1]. An estimated 60 to 80 million couples suffer from infertility [2, 3]. Infertility is a devastating experience for both the genders [4]. It can lead to marital conflict, hopelessness, guilt, shame, worthlessness, anxiety, depression, social isolation, sexual dysfunction, and decreased sexual self-esteem [5, 6].

Studies conducted in South Asian low and middle income countries (LMIC) report that 35 to 50% infertility is due to “male factor” infertility [7, 8], but, unfortunately social and cultural factors influence the perception of the society. In a patriarchal society like ours, child bearing inability is often attributed to female partners solely who suffer humiliation, and discrimination [1, 9–12].

Consequently, infertile women are omitted from social and traditional rituals [13], they are abused physically, emotionally, and verbally, and often end up getting divorced [12, 13]. Thus, women who are blamed for childlessness suffer from personal grief, frustration, poor mental health and social dis-functioning which leads to negative impact on their psychological health [14–17].

Fertility also affects an individual’s quality of life (QoL). The World Health Organization (WHO) [18] defines QoL as an “individuals’ perceptions of their position in life in the context of the culture and value systems in which they live and in relation to their goals, expectations, standards, and concerns” Several studies have identified that infertility can lead to poorer QoL [11, 19, 20]. However, depression, anxiety, suicidal ideation, and poor quality of life are exhibited more in infertile women as compared to infertile men [11, 13]. Studies from India, Italy, China, and Iran indicate that infertility in female partners has negative impact on their marital life [6, 11, 21].

However, studies from Bangladesh and India suggest that infertile males are also stigmatised, socially disgraced, and their manhood is questioned, resulting in their reluctance to seek treatment [22, 23].. Although the psychological burden due to infertility is high, there is inconsistency in the reported literature [15], as not all couples report being socially distressed; their own resilience and social support seem to play a vital role in their emotional stability [24].

Resilience can act as a buffer against the negative psychological impact of infertility and help develop psychological tolerance. Individuals having resilience have high self-esteem, optimism, self-confidence, problem solving abilities, and life satisfaction. Thus, resilience is, apparently, the key to improving the QoL among infertile males and females [15, 20].. Therefore, there is dire need to explore the quality of life of infertile males and females from the cultural context of Pakistan and to explore its undesirable psychological outcomes. Moreover, it is also imperative to explore the coping mechanisms among infertile patients, which can ultimately prevent them from developing mental illnesses. Thus, in the light of literature, the objectives of this study were:

1. To compare the QoL of infertile males and females presenting at the infertility centre for treatment.
2. To assess its association of QoL with resilience, depression, and other socio-demographic factors in males and females presenting at the infertility center for treatment.

## Methods

An analytical cross sectional study was conducted, at the Australian Concept Infertility Medical Centre (ACIMC), Karachi, Pakistan. The rationale behind the selection of this study Centre was that ACIMC is the only Centre in Karachi having the maximum flow of infertile couples seeking infertility treatment, as compared to the other infertility Centres. The additional benefit of selecting this Centre was that it had representation from all ethnic and socio-economic groups, as infertile couples are referred here from the seven sub branches of the Centre located in different provinces of Pakistan; moreover, the Angel Trust caters to the needs of the non-affording.

All Pakistani infertile men and women seeking fertility treatment, and who gave written consent, were included in this study. Patients with any known case of psychiatric illness were excluded. Our national language is Urdu which is spoken by all the ethnicities along with their own regional language. However, we excluded those who could not converse in the Urdu language because our questionnaire was in Urdu.

Purposive sampling technique was employed for selecting the participants. The infertile males and females who were receiving treatment for infertility at AIMC as per their scheduled appointment were approached by the trained data collector. The participants were screened for eligibility and those who gave written consent for participation, were enrolled in the study. This study was the secondary objective of our original project, whose primary objective was to determine the association of marital maladjustment with quality of life, depression, and resilience among infertile couples. Thus, the sample size (*n* = 668) for this study was achieved on the basis of our primary objective (paper in press), considering 80% power, significance level 5%, attrition rate 10%, and the anticipated odds ratio of 2 [10, 15, 25–29].

### Outcome variable

#### Quality of life

The FertiQoL tool was used to assess the quality of life of infertile patients. This tool was validated in Urdu in our previous study (paper in press). The FertiQoL questionnaire is a self-reporting questionnaire, [30] specifically designed by experts from the European Society of Human Reproduction and Embryology (ESHRE) and the American Society of Reproductive Medicine (ASRM) to assess the Quality of Life of infertile patients.. It comprises of two modules: Core FertiQoL and Treatment FertiQoL module. The Core FertiQoL consists of 24 items, categorized into four domains: emotional, cognitive and physical (marked as mind/body), relational, and social. The Treatment FertiQoL module, is an optional treatment module, consists of 10 items, which are further categorized into two domains: environment and tolerability for the treatment for infertility. The scale ranges from 0 to 4, and a higher score indicates better QoL.

### Independent variables

#### Depression

Depression was assessed through the Urdu version of the Beck II Depression Inventory Tool, (the content and face validity was performed) [25, 31, 32]. This tool covers a broad behavioural spectrum and is easy to understand therefore is widely used in Pakistan. It is a 21 item self-reporting measure of depressive symptomatology, which includes: sadness, pessimism, past failure, loss of pleasure, guilty feelings, punishment feelings, self-dislike, self-criticalness, suicidal thoughts or wishes, crying, agitation, loss of interest, indecisiveness, worthlessness, loss of energy, changes in sleeping pattern, irritability, changes in appetite, concentration difficulty, tiredness or fatigue, and loss of interest in sex. The recommended cut off score for depression is 14. The higher the score the greater the severity of depressive symptoms. This tool has been widely used in Pakistan. Many AKU initiated research projects have used the tool because it covers a broad behavioural spectrum and is easy to understand.

#### Resilience

Resilience, is the ability to rebound or spring back, in other words the power to resume to its original shape or position after compression or bending [33]. Data for resilience was collected through the validated Urdu version of Resilience Scale 14 (RS-14) [31]. Participants were considered resilient, if they scored atleast 73 on the RS-14 scale.

#### Socio-demographic factors

A structured questionnaire was administered to obtain preliminary information about participants age, education, language, number of family members, type of marriage, its duration, personal health; reproductive history, (including number of miscarriages, alive/dead children, age of last child for secondary infertility cases), cause of infertility (“male factor”/female), extra marital affairs or/and multiple marriages; and social and religious support mechanisms.

### Ethical approval

The ethical approval (ERC No. 4615-SON-ERC-17) was received from the Institutional Ethical Review Committee, AKU; and approval was also sought from the participating study site, AIMC. A written informed consent form, signed by the study participants, was obtained before the initiation of this study. We assured complete confidentiality of the study participants. The data was only accessible to the researchers and the responses were reported in group form; no individual case was identified.

### Plan of analysis/data management

The data was double entered in Microsoft access software by trained data entry operators. The overall quality of the study was maintained through random spot-checks. The data base was pass word protected and was only accessible by the research group. Missing data was dealt by imputation. Statistical analysis was done on STATA version 12. Descriptive analyses for quantitative variables were reported as mean ± SD/ median (IQR) and were assessed through the t test or the Mann Whitney test, as deemed appropriate. Frequency and percentages were reported for qualitative variables and were assessed by the chi square test or the fisher exact test, as deemed appropriate. Unadjusted and adjusted beta coefficients, along with their 95% CI, were reported using linear regression analysis, to determine the association of resilience, depression, and other factors with the total QoL of males and females, by. All plausible interactions and confounders were assessed. A *p* - value of < 0.05 was considered as significant.

## Results

In all, 334 males and 334 females, presenting at the infertility centre for treatment, were enrolled. 584 (87.4%) had primary infertility and 84% (12.6%) had secondary infertility. A higher proportion 148 (22%) had male factor infertility, 130 (19.5%) had female factor infertility, 10 (1.5%) had both male and female factor infertility, and 56.9% didnot know the cause.

### Socio-demographic factors of the study participants

Table 1 is divided into 3 sections: Demographic factors, Socio-economic factors, and Social religious network of the infertile patients presenting at the infertility centre.

**Table 1:** Socio-demographic factors of the study participants presenting to the infertility Centre

	Male (***n*** = 334)	Female (***n*** = 334)	***P*** value
**A. Demographics**			
**Age (in years)**			
Mean ± SD	35.53 ± 6.72	30.87 ± 6.12	0.001*
**Formal Education**			
Yes	311 (93.1%)	281 (84.1%)	< 0.001*
No	23 (6.9%)	53 (15.9%)	
**Years of education (in years)**			
Median (IQR)	14 (10–16)	12 (7–16)	0.001*
**Informal Education**			
Yes	167 (50.0%)	209 (62.6%)	0.001*
No	167 (50.0%)	125 (37.4%)	
**Role in the family**			
Head	156 (46.7%)	8 (2.40%)	< 0.001*
Not Head but take part in decision	171 (51.2%)	251 (75.1%)	
Does not take decision, only follower	7 (2.10%)	75 (22.5%)	
**First Marriage**			
Yes	305 (91.3%)	321 (96.1%)	0.011*
No	29 (8.70%)	13 (3.90%)	
**B. Socioeconomic**			
**Working**			
Yes	326 (97.6%)	52 (15.6%)	< 0.001*
No	8 (2.40%)	282 (84.4%)	
**Spouse employed**			
Yes	50 (15.0%)	330 (98.8%)	< 0.001*
No	284 (85.0%)	4 (1.20%)	
**Total household income (in PKR)** **Median (IQR)**	50,000 (30000–90,000)	35,000 (20000–50,000)	< 0.001*
**Total Monthly income (in PKR)**			
1000–25,000	61 (18.4%)	84 (25.3%)	< 0.001*
25,000–40,000	58 (17.5%)	86 (25.9%)	
40,000–80,000	106 (32.0%)	103 (31.0%)	
80,000–10,000,000	106 (32.0%)	59 (17.8%)	
**Total**	331	332	
**C. Social/Religious Network**			
**No of meet up with friends/week**			
< 1 times /week	195 (58.4%)	265 (79.3%)	< 0.001*
1–5 times/ week	133 (39.8%)	49 (14.7%)	
≥ 5 times/week	6 (12.3%)	6 (3.40%)	
**Religious activities**			
Yes	310 (92.8%)	331 (99.1%)	< 0.001*
No	24 (7.20%)	3 (0.90%)	

*significant at *p* value< 0.05 by t test/chisquare/fisher exact test

Section A of Table 1 describes the demographic factors of the study participants. The mean age was significantly higher among males, 35.53 ± 6.72, as compared to females, 30.87 ± 6.12 (*p* value 0.001). A higher proportion of males (93.1%) had formal education as compared to their counterparts (84.19%) (*p* -value < 0.001), with higher median years of education among males as compared to females. However, a higher proportion of females (62.6%) had informal education as compared to their counterparts (50%) (*p* value = 0.001). We also observed that a significantly higher proportion of males (46.7%) were heads of the family as compared to females (2.4%) (*p* value < 0.001). Moreover, a significantly higher proportion of males (8.7%) had more than one marriage as compared to (3.9%) females who were previously married.

Section B of Table 1 describes the socio-economic status of the study participants. We observed that a significantly higher proportion of females (84.4%) were not working as compared to males (2.4%) (*p* value < 0.001). The median monthly household income reported by males was significantly higher, i.e. PKR 50,000 (30,000–90,000), as compared to females, PKR 35,000 (20,000–50,000) (*p* value < 0.001).

Section C of Table 1 presents the social/ religious network of the study participants. We observed that the males had a greater number of meet ups with their friends as compared to females (*p* value < 0.001). Moreover, we observed that a significantly higher proportion of females (99.1%) were involved in religious activities as compared to males (92.8%) (*p* value < 0.001).

### Resilience, depression, and QoL in infertile males and females

Table 2 shows resilience, depression, and QoL in infertile males and females. We observed that the mean resilience scores were significantly higher among males, 77.64 ± 8.56, as compared to females, 76.19 ± 8.69 with a 95% CI; − 2.757, − 0.1347 (*p* value = 0.031). The proportion of less resilient females (29.6%) was significantly higher than that of less resilient males (21.3%). However, a significantly higher proportion of females were depressed (13.8%) as compared to males (6%). We observed that the mean QoL scores for the general health domain, emotional domain, mind and body domain, and relational domain, and the total QoL were significantly higher in males as compared to females (*p* value< 0.001); however, QoL for the social domain was not significantly different in both the groups.

**Table 2:** Resilience, Depression and QoL among infertile males and females

Resilience/Depression	Male (***n*** = 334)	Female (***n*** = 334)	***p***-value
**Resilience**			
**Resilience (Mean ± SD)**	77.64 ± 8.56	76.19 ± 8.69	0.031*
**Resilience**			0.013*
**< 73 (less resilient)**	71 (21.3%)	99 (29.6%)	
**≥ 73 (more resilient)**	263 (78.7%)	235 (70.4%)	
**Depression**			
**Depression (Median (IQR))**	3.00 (1.00–7.00)	7.00 (2.00–12.00)	< 0.001*
**Depression**			< 0.001*
**< 17 (not depressed)**	314 (94.0%)	288 (86.2%)	
**≥ 17 (depressed)**	20 (6.0%)	46 (13.8%)	
**Quality of life**			
**General Health** Mean ± SD	56.45 ± 19.36	48.34 ± 11.52	< 0.001*
**Emotional Domain** Mean ± SD	82.63 ± 13.43	60.02 ± 23.38	< 0.001*
**Mind and Body Domain** Mean ± SD	85.65 ± 15.46	55.40 ± 23.60	< 0.001*
**Relational Domain** Mean ± SD	79.98 ± 19.56	88.76 ± 10.60	< 0.001*
**Social Domain** Mean ± SD	78.23 ± 13.35	77.75 ± 18.05	0.696
**Total Qol scores** Mean ± SD	81.58 ± 12.15	70.48 ± 15.69	< 0.001*

*significant at *p* value < 0.05 by chi-square of independence/ t test

### Univariate analysis to assess the relationship of depression, resilience, and demographic factors with the total quality of life, in males and females presenting for infertility treatment

Table 3 presents the univariate analysis to assess the relationship of demographic factors, with the total quality of life, in males and females presenting for infertility treatment.

**Table 3:** Univariate analysis to assess relationship of demographic factors with total quality of life among males and females presenting for infertility treatment

Variables	Univariate analysis			
	Males Unadjusted beta coefficient (SE)	95% CI	Females Unadjusted beta coefficient (SE)	95% CI
**Age (in years)**	−0.043 (0.099)	− 0.239, 0.151	− 0.054 (0.140)	− 0.331, 0.221
**Formal Education**				
Yes (ref)				
No	−9.051 (2.569)	−14.106, −3.996*	−6.101 (2.329)	− 10.683, −1.518*
**Years of formal education (in years)**	0.269 (0.070)	0.131, 0.407 *	0.135 (0.071)	−0.004, 0.275*
**Informal Education**				
Yes (ref)				
No	2.345 (1.319)	−0.249, 4.940*	3.113 (1.768)	−0.365, 6.592*
**Type of Marriage**				
Self-Choice (ref)				
Arranged	0.119 (1.611)	−3.051, 3.289	−6.122 (2.277)	−10.602, −1.642*
**Duration of marriage (in years)**	−0.302 (0.124)	−0.546, −0.057*	0.051 (0.139)	−0.221, 0.324
**Type of family**				
Extended	2.176 (1.378)	−0.535, 4.888*	2.759 (1.799)	−0.781, 6.300*
Nuclear (ref)				
**Role in the family**				
Head (ref)				
Not Head but take part in decision	−0.449 (1.340)	−3.086, 2.186*	−11.740 (5.565)	−22.629, − 0.730
Does not take decision, only follower	−5.367 (4.677)	− 14.568, 3.833	− 16.373 (5.763)	− 27.711, − 5.036
**Type of infertility**				
Primary	−1.585 (1.976)	−0.547, 2.303	−9.248 (2.571)	−14.305, −4.191*
Secondary (ref)				

*significant at p value < 0.25 by univariate analysis

We observed that age had a significant association with Qol of male and female infertile patients. However, male and females with no formal education had significantly lower Qol scores i.e. 9 and 6 units, respectively, as compared to those with formal education. Similarly, years of formal education significantly increased the QoL of males and females. The QoL scores of female patients who had arrange marriage was 6 units significantly lower as compared to those who had love marriage. On the other hand, there was no significant association in the type of marriage and QoL scores among males. However, there was significant negative association of duration of marriage with QoL among males, but not among females. Moreover, males and females who lived in extended families had significantly higher QoL scores as compared to those who lived in nuclear families. Furthermore, the QoL scores were significantly higher of females who were not the head of the family but took part in decision making as compared to those who did not take part in decision making. The QoL of females with primary infertility was 9 units significantly lower as compared to those with secondary infertility, however there was no significant association of QoL and type of infertility among males.

We also evaluated the relationship of socio-economic factors with mean QoL in males and females (Table 4). We did not observe any significant association of QoL with working status in males and females. However, QoL of females, working outside their house was 9 units significantly higher compared to those working from home. Moreover, the QoL was significantly lower among males and females with low total household monthly income. Furthermore, QoL of males and females who did not have a television and/or a refrigerator in their house, their own cultivated land, and a vehicle was significantly low as compared to those who had any or all of these. Additionally, the quality of life of males decreased significantly by 0.6 units with increase in number of friends; however, this did not have any significant relationship with the QoL of females.

**Table 4:** Univariate analysis to assess relationship of Socioeconomic with total quality of life among males and females presenting for infertility treatment

Variables	Males Unadjusted beta coefficient (SE)	95% CI	Females Unadjusted beta coefficient (SE)	95% CI
**Working status**				
Yes (ref)				
No	−4.957 (4.325)	−13.467,3.5523	− 0.319 (2.371)	−4.984, 4.346
**Work Place**				
Inside the house (ref)	–	–	–	–
Outside the house	−4.418 (1.829)	−8.016,-0.820*	9.143 (3.908)	1.288, 16.997*
Both	9.312 (12.015)	−14.325,32.950	−13.706 (9.972)	−33.746, 6.334
**Total Monthly income (in PKR)**				
1000–25,000	−6.385 (1.921)	−10.165, −2.604*	−8.401 (2.582)	− 13.479, − 3.322*
25,000–40,000	−2.255 (1.952)	− 6.097, 1.586	0.009 (2.569)	−5.045, 5.063
40,000–80,000	−4.056 (1.646)	−7.295, − 0.817*	2.499 (2.477)	− 2.373, 7.373
80,000–10,000,000(ref)	–	–	–	–
**TV in the house**				
Yes (ref)	–	–	–	–
No	−4.945 (1.941)	−8.763, −1.128*	−6.244 (2.471)	− 11.161, − 1.382*
**Refrigerator in the house**				
Yes (ref)	–	–	–	
No	−6.655 (2.106)	−10.797, −2.513*	− 10.016 (2.912)	−15.744, −4.287*
**Own cultivated land**				
Yes (ref)	–	–	–	–
No	−4.184 (1.658)	−7.446, −0.923*	−5.092 (1.954)	−8.937, −1.248*
**Own Vehicle**				
Yes (ref)	–	–	–	–
No	−6.914 (1.575)	−10.011,-3.815*	−6.760 (1.989)	−10.673,-2.847*
**Number of friends**	−0.603 (0.202)	−0.1000, − 0.206*	−0.126 (0.320)	− 0.757, 0.504

*significant at *p* value < 0.25 by univariate analysis

We also evaluated the relationship of resilience and depression with QoL in males and females (Table 5) and we observed that QoL of males and females who had low resilience was 12 and 13 units significantly lower, respectively, as compared to those who had higher resilience. Moreover, QoL in males and females was 21 and 22 units significantly lower, respectively, among those were depressed as compared to those who were not depressed.

**Table 5:** Univariate analysis to assess relationship of Resilience and Depression with quality of life among males and females presenting for infertility treatment

Variables	Males Unadjusted beta coefficient (SE)	95% CI	Females Unadjusted beta coefficient (SE)	95% CI
**Resilience**	0.677 (0.067)	0.543,0.811*	0.828 (0.088)	0.655,1.001*
**Resilience**				
< 73 (less resilient)	−12.018 (1.479)	− 14.92, −9.108*	−13.278 (1.736)	− 16.694,-9.863*
≥ 73 (more resilient) (ref)	–	–	–	–
**Depression**	−1.057 (0.092)	−1.238, − 0.888*	−1.597 (0.082)	− 1.758, − 1.435*
**Depression**				
< 17 (not depressed) (ref)	–	–	–	–
≥ 17 (depressed	−21.490 (2.532)	−26.471,16.509*	−22.369 (2.172)	− 26.642, 18.095*

*significant at *p* value < 0.25 by univariate analysis

### Multivariable analysis to assess the relationship of depression, resilience, and demographic factors with the total quality of life, in males and females presenting for infertility treatment

Table 6 shows the multivariable analysis to assess the relationship of demographic factors, socio-economic factors, resilience, and depression with the total quality of life, in males and females presenting for infertility treatment.

**Table 6:** Multivariable analysis to assess relationship of depression, resilience and demographic factors with total quality of life among males and females presenting for infertility treatment

Variables	Multivariable analysis			
	Males Adjusted Beta Coefficient (SE)	95% CI	Females Adjusted Beta Coefficient (SE)	95% CI
**Resilience**				
< 73 (less resilient)	−8.470 (1.422)	−11.268,-5.672*	−8.606 (1.599)	−11.753, −5.458*
≥ 73 (more resilient) (ref)	–	–	–	–
**Depression**				
< 17 (not depressed) (ref)	–	–	–	–
≥ 17 (depressed)	−17.849 (2.365)	−22.503, − 13.196*	−19.387 (2.078)	− 23.476, − 15.298*
**Formal Education**				
Yes (ref)	–	–	NS	NS
No	−5.374 (2.245)	−9.794, −0.954*		
**Number of friends**	−0.554 (0.172)	− 0.893, − 0.216*	NS	NS
**Total Monthly income (in PKR)**				
10,000-25,000	−3.551 (1.687)	−6.870,-0.231*	−7.249 (2.161)	−11.501, −2.996*
25,000–40,000	−1.793 (1.670)	−5.079,-1.493	−2.615 (2.155)	−6.854, 1.624
40,000–80,000	−2.747 (1.386)	−5.474,-0.020*	−0.644 (2.078)	−3.443, 4.732
80,000–10,000,000(ref)	–	–	–	–

*Significant at *p* value < 0.05 by multivariable analysis

*NS* non significant

We observed that among males resilience and depression had a significant association with QoL, after adjusting for the covariates educational status, monthly income, and number of friends. Males who were less resilient their QoL was 8 units significantly lower as compared to those who were more resilient. Similarly, those males who were depressed their QoL was 17 units significantly lower as compared to those who were not. Moreover, males who had no formal education their QoL was 5 units lower as compared to those who had received formal education. Males whose household monthly income was between 10,000–80,000 PKR their QoL was lower as compared to those who had an income between 80,000-10,000,000 PKR. Furthermore, males who had more friends had lower QoL scores.

We observed that among females resilience and depression had a significant association with QoL, after adjusting for the covariate monthly income. Females who were less resilient their QoL was 8 units lower as compared those who were more resilient. Similarly, those females who were depressed their QoL was 19 units significantly lower as compared to those who were not. Moreover, females whose household monthly income was between 10,000–25,000 PKR their QoL was 7 units significantly lower as compared to those who had an income between 80,000-10,000,000 PKR.

## Discussion

This study aimed to compare the QoL of males and females presenting at the infertility centre for treatment and to assess its association with other factors.

This study showed that infertile males were more resilient than infertile females. The plausible reasons for men being more resilient than females in Pakistan would be our society norms, where men are treated as superiors and have more rights, power, and authority to take decisions.

Moreover, in a developing country like ours, that has a patriarchal and polygamous society, married women who are unable to conceive are stigmatized and blamed by their spouses and in-laws, which leads to depression among infertile females, and high prevalence of depression is significantly associated with low QoL. This finding is consistent with other studies that were carried out in Ghana, Iraq, and Germany, which also indicated that females scored high in depression. A study carried out in western Iran also found that 76% of infertile women suffered from depression, while 61.5% suffered from clinical depression [34–36]. Another study showed that prevalence of depression and anxiety in infertile women was high as compared to men [6]. Moreover, the divorce rate is twice as high amongst infertile couples and the fear of remarriage of their husbands adds to their misery [37].

Furthermore, females undergo numerous invasive procedures for infertility diagnosis and treatment, in comparison to males [21]. All these factors have a negative impact on the females, contributing to the high prevalence of depression among them. Our findings are comparable to those reported by infertile women in developing countries, including Iran, Taiwan, India, Tunisia, and China, and developed countries, like the USA, Poland, and Italy [38–44]. However, a few studies, have not found any association between QoL and depression among infertile women [45, 46].

In our study, the infertile females and males having higher household monthly income had a significantly better QoL in comparison to those with lower monthly income. This is comparable to the study carried out in Iran, by Namdar et al. [47], which also reported a positive association between monthly income and QoL in women. Another study conducted in Iran on infertile couples also reported a positive association, which is consistent with our findings, that higher income is associated with better QoL among the male gender [48]. Hence, this shows that financial stability can compensate for the compromised quality of life of an infertile individual. A reason for this could be that infertility treatment is expensive, therefore, individuals with financial instability possibly feel more distressed due to the high cost of the treatment, versus those who are financially stable.

In addition, our study identified that infertile females and males having higher resilience had a significantly better QoL in comparison to those with low resilience scores. Literature also portrays that resilience is positively associated with fertility QoL. Resilience also has a moderating effect on psychological stress associated with QoL ([20]. Moreover, another study also supports that resilience is a protective factor against infertility related distress and impaired quality of life for infertile couples [15].

Our study results showed that infertile males having a large circle of friends had lower QoL scores. Presumably, with more friends the infertile males may face more social pressure or a lot of probing questions, which adds to their frustration related to infertility, resulting in lower QoL scores. Literature also suggests that men have less social support and are less likely to confide in friends about infertility as compared to women [49]. On the other hand, a reason why infertile males have a growing social circle of friends could be that this serves as a diversion, or as a coping mechanism, or it provides an outlet for their emotions to overcome the feelings of hopelessness. Our findings are comparable with the results of another study [50]. However, no association was found between social circle and QoL among infertile females. But, Steuber et al. [51] report that women who are unable to disclose their infertility have poor QoL due to unmet social support.

Another finding of the present study was that males with a lower educational level had low QoL scores. This finding is in agreement with the findings of Jahromi et al. and Drozdzol et al., which highlight that infertile men with low or no academic education had lower QoL scores [11, 52].

The current study had several strengths and limitations. This is probably the first study to describe the association of QoL with resilience, depression, and other socio-demographic factors in infertile males and females at an infertility clinic. Moreover ACIMC in Karachi caters to diverse socio-economic and ethnic groups as patients are referred here from seven sub-branches of the Centre located in different provinces of Pakistan. Additionally the Trust at the centre caters to the needs of the non-affording, thus uplifting the generalizability of our study.. Contextual and reliable tools were used to assess QoL with resilience, depression, and other factors in infertile males and females.

However, the limitations of our study were that data on QoL and depression was collected using self-reporting tools; hence, the element of reporting bias could not be eliminated; though, special attention was given to ensure the privacy and confidentiality of the data collection process, to minimize this effect. Moreover, it was a single-centred study, hence, the results cannot be generalized to all infertile individuals residing in the communities and not utilizing any treatment, but it can be generalized to all private infertility clinics similar to our setting. In addition, we have not explored two important variables i.e. drug use/ abuse and other type of comorbidities such as endometriosis etc. so we cannot generalize our results on these women/ men.

## Conclusion

This study concluded that amongst infertile men QoL was negatively associated with no formal education, number of friends and depression; however, it had a positive association with income and resilience. Among females, QoL was associated with depression, resilience, and monthly income. Hence, future studies are required to explore the effectiveness of gender-specific mental health interventions, such as resilience building, to decrease depression and improve QoL among infertile individuals. Moreover, mental health experts, fertility experts, and other health care providers must be adequately trained to respond to the holistic needs of individuals going through fertility related problems.

## Data Availability

The dataset used in current study is available from the corresponding author and can be released upon reasonable request.

## Abbreviations

ACIMC: Australian Concept Infertility Medical Centre
LMIC: Low Middle Income Country
QoL: Quality of Life
AKU: Aga Khan University
ESHRE: European Society of Human Reproduction and Embryology
ASRM: American Society of Reproductive Medicine
SD: Standard Deviation
IQR: Inter quartile range
